# Cloning, Structural Characterization, and Phylogenetic Analysis of Flower MADS-Box Genes from Crocus (*Crocus sativus* L.)

**DOI:** 10.1100/tsw.2007.175

**Published:** 2007-06-22

**Authors:** Athanasios S. Tsaftaris, Alexios N. Polidoros, Konstantinos Pasentsis, Apostolos Kalivas

**Affiliations:** ^1^ Institute of Agrobiotechnology, Center for Research and Technology Hellas, Thermi, GR-570 01, Greece; ^2^ Department of Genetics and Plant Breeding, Aristotle University of Thessalonik, Thessaloniki, GR-541 24, Greece

**Keywords:** Crocus sativus L., monocots, MADS-box genes

## Abstract

Crocus (*Crocus sativus* L.) is a crop species cultivated for its flowers and, more specifically, for its red stigmas. The flower of crocus is bisexual and sterile, since crocus is a triploid species. Its perianth consists of six petaloid tepals: three tepals in whorl 1 (outer tepals) and three tepals in whorl 2 (inner tepals). The androecium consists of three distinct stamens and the gynoecium consists of a single compound pistil with three carpels, a single three-branched style, and an inferior ovary. The dry form of the stigmas constitutes the commercial saffron used as a food additive, in the coloring industry, and in medicine. In order to uncover and understand the molecular mechanisms controlling flower development in cultivated crocus and its relative wild progenitor species, and characterize a number of crocus flower mutants, we have cloned and characterized different, full-length, cDNA sequences encoding MADS-box transcription factor proteins involved in flower formation.

Here we review the different methods followed or developed for obtaining these sequences involving conventional 5' 3' RACE, as well as newly developed methods from our group, named Rolling Circle Amplification – RACE (RCA-RACE) and its modification named familyRCA-RACE (famRCA-RACE). Furthermore, the characteristics of the protein structure and their common and specific domains for each type of MADS-box transcription factors in this lower nongrass monocot belonging to the Iridaceae family are described. Finally, a phylogenetic tree of all the MADS-box sequences available in our lab is presented and discussed in relation to other data from studies of species of the Iridaceae group and closely related families from an evolutionary perspective. The structural and phylogenetic analyses are based on both published and unpublished data.

## INTRODUCTION

Flowering plants represent one of the most successful and diverse groups of organisms on the planet, with more than 250,000 extant species in the wild and many more varieties generated by horticulturists through hybridization and other breeding efforts. The majority of flowers possess four types of floral organs: two outer whorls of sterile organs, the sepals and petals (also known as the perianth), and two inner whorls of fertile organs, the male stamens and female carpels, with the carpels positioned centrally. Although the fundamental characteristics of angiosperm flowers are generally conserved, the enormous morphological diversity suggests a high degree of plasticity in the genetic control of floral development. Variation is observed in every aspect of floral architecture, including phyllotaxy, merosity, floral symmetry, and floral organ identity. In-depth analyses of model species, such as *Arabidopsis thaliana* and *Antirrhinum majus,* have contributed significantly to our understanding of the genetic pathways that control these morphological components. By using this work as a foundation for comparative studies, a picture is gradually coming into focus of how alterations in floral genetic programs have contributed to the evolution of floral architecture.

Forward mutagenesis studies of these two model species uncovered an intriguing series of homeotic floral mutants[1,2,3]. In both taxa, the mutants appeared to fall into similar classes: mutations that affected sepal and petal identity were placed into what was termed the “A”class; those that affected petal and stamen identity, the “B” class; and those that affected stamen and carpel identity, the “C” class. For instance, B mutants exhibited the transformation of petals into sepals and stamens in carpels[2,4]. Analysis of double and triple mutants[5] led to the proposition of a simple and elegant model that explained the major aspects of genetic interactions among the loci. This became known as the ABC model[3]. Fundamentally, the ABC model holds that the overlapping domains of three classes of gene activity, referred to as A, B, and C, produce a combinatorial code that determines floral organ identity in successive whorls of the developing flower. Another critical component of the ABC program is that A and C functions are mutually exclusive[5], such that elimination of C gene activity causes the A domain to expand and vice versa[6,7]. Additional studies of mutants and overexpressing lines have largely confirmed the model and demonstrated the completely homeotic nature of this developmental program[5,8,9]. As our understanding of the genes involved in the program has grown, the model has expanded as well to what is now referred to as the ABCDE model[10]. “D” class genes were proposed as ovule-identity genes based on work done in *Petunia hybrida*[11], while “E” class genes function broadly across the floral meristem to facilitate the function of many of the original ABC loci[12,13]. With the exception of AP2, all of the organ-identity genes identified to date are members of the paneukaryotic MADS transcription factor family[14,15]. MADS-box genes are characterized by the highly conserved MADS-box domain and can be divided into the type I and type II main lineages that are present in plants, animals, and fungi[16,17]. These lineages differ in the amino acid sequence of the MADS-box as well as in the domain structure of the predicted protein. Most type II proteins exhibit a typical MIKC structure, where the MADS domain is followed by a short I (intervening) domain, a well-conserved K (keratin-like) domain, and a variable C-terminal region, while type I proteins lack the K domain, forming a structure of a MADS-box followed by a rather undefined and length-variable C domain[17].

Almost all the MIKC-type MADS-box genes of plants can be subdivided into 12 major clades, each of which includes one to six paralogs from *Arabidopsis* and putative orthologs from other seed plants[14]. Genes of the different A, B, C, D, and E classes related to flower development fall into separate phylogenetic clades that are known as APETALA1/FRUITFULL (AP1/FUL) or SQUAMOSA (SQUA) (class A), APETALA3 (AP3) and PISTILLATA (PI) or DEFICIENS (DEF) and GLOBOSA[18] (class B), AGAMOUS (AG) (classes C and D), and AGAMOUS-like6 (AGL6) and SEPALLATA (SEP) (class E)[14]. Not all the genes in the above-mentioned clades determine floral organ identity. Genes from other clades may be related to flowering as well through their function in determining flower initiation and meristem identity. These are the FLOWERING LOCUS C-like (FLC-like) clade with the *Arabidopsis* genes FLC and AGL27 that are inhibitors of flowering, the STMADS11-like clade with the genes AGL24 and SHORT VEGETATIVE PHASE (SVP) that are repressors of flowering in vegetative tissues, and the Tomato MADS-box gene 3-like (TM3-like) clade with SUPPRESSOR OF OVEREXPRESSION OF CONSTANS 1 (SOC1) that is involved in regulation of flowering time[14].

Although monocot flowers contain stamens and carpels, they differ from eudicot flowers in the type of organs that are present in the outer whorls. Liliaceae family members often have two outer whorls of showy petal-like tepal organs, whereas grass flowers have paleas, lemmas, and lodicules instead of sepals and petals. A modified ABC model in which a B function is present in whorls 1, 2, and 3 has been proposed to explain the presence of tepals in Liliaceae flowers[19].

Studying flower development is not only significant for improving our understanding of basic regulatory mechanisms of flower initiation and organ identity, but could have practical applications in crops cultivated for their flowers. Crocus is an example of such a crop with flowers of economic importance, and we have set up a study to understand and possibly improve the crocus flower. Crocus is a monocot triploid sterile species belonging to the Iridaceae family, whose red stigmatic styles constitute saffron, a popular food additive with a delicate aroma and attractive color. Saffron also has medicinal properties and is used in the coloring industry. Different wild-type species like *Crocus cartwrighianus*, *C. hadriaticus,* and *C. oreoreticus* have been proposed as progenitor species, giving rise to the triploid sterile cultivated *C. sativus*, but no conclusive molecular data have been obtained, yet, to identify the two progenitor species with certainty.

The flower of crocus is bisexual and sterile. The perianth consists of six petaloid tepals: three tepals in whorl 1 (outer tepals) and three tepals in whorl 2 (inner tepals). The androecium consists of three distinct stamens and the gynoecium consists of a single compound pistil with three carpels, a single three-branched style, and an inferior ovary. Several phenotypic flower mutants have been described, such as flowers with larger numbers of styles and stamens as well as flowers without stamens[20,21]. Crocus blooms only once a year and is hand harvested. After mechanical separation of the tepals, the stigmas are hand separated from the carpels and dried. The size and the amount of individual stigmas collected from each flower influence the total yield and quality of saffron. Between 70,000 and 200,000 flowers are needed to produce 1 kg of dried saffron, which equates to 370–470 h of work. Consequently, the cultivation of this crop for its flowers and specifically its stigmas is very labor intensive, leading to high costs[22]. Thus, understanding flower development in crocus could reveal ways to increase yield and lower production costs since flowers and, more specifically, isolated stigmas comprise the valuable commercial part of the plant. With this goal, we have cloned, characterized, and studied the expression of all types of MADS-box genes from crocus involved in flower formation. Obtaining a similar type of molecular information for the three putative progenitor species, *C. cartwrighianus*, *C. hadriaticus,* and *C. oreoreticus,* and comparing them with the cultivated *C. sativus* genes, could contribute to the identification of its diploid progenitor species.

The different methods used to clone the sequences of MADS-box genes expressed in the flower of crocus are reviewed and their comparative structural and phylogenetic relationships are described.

## CLONING THE MADS-BOX SEQUENCES FROM CROCUS

### The Use of Conventional 5′ 3′ RACE

Table 1 displays homologues of the major A, B, C, D, and E types of MADS-box genes from crocus; the two dicot model species, *Arabidopsis* and *Antirrhinum*; and the monocot grass model, crop rice. The number of homologues for each type of MADS-box sequences obtained from crocus and the methods followed to obtain each sequence are also indicated.

In their recent analysis of all the MADS-box genes of *Arabidopsis* flower, Wellmer et al.[23] found that 17 out of 39 MIKC-type MADS-box genes were shown to be up-regulated in early flower development. We do not know how many MADS-box genes exist in the crocus genome, but as it is an allotriploid species[24], we expect this number to be much higher in crocus. Isolation of full-length gene transcripts is important to determine the protein-coding region and study gene structure. However, isolation of novel gene sequences is often limited to expressed sequence tags (ESTs) (i.e., short cDNA fragments that predominantly represent the 3' end of the transcript). Rapid amplification of cDNA ends

(RACE) is by far the most popular approach for obtaining full-length cDNAs when only part of the transcript's sequence is known. Since its original description[25,26], numerous modifications and

**TABLE 1**
**Homologues of the Major A, B, C, D, E Types of MADs-box Genes from Crocus, the Two Dicots Model Species *Arabidopsis* and *Antirrhinum* and the Monocot Model, Rice*.**

| Gene class | Arabidopsis | Antirrhinum | Oryza | Crocus | Number of Crocus homologues sequenced | Cloning method (Crocus) | Reference (Crocus) |
|---|---|---|---|---|---|---|---|
| A | *APETALA1* | *SQUAMOSA* | *RAP1* | *CsatAP1/FUL* | 3<br>2 | 5′ 3′ RACE and famRCA-RACE | [22]<br>[39] |
| B | *APETALA3*<br>*PISTILLATA* | *DEFICIENS*<br>*GLOBOSA* | *SUPERWOMAN 1*<br>*OsMADS2, OsMADS4* | *CsatAP3*<br>*CsatPI* | 2<br>5 | 5′ 3′ RACE<br>5′ 3′ RACE | [27]<br>[29] |
| C | *AGAMOUS* | *FARINELI* | *OsMADS3* | *CsatAG* | 1 | 5′ 3′ RACE | [28] |
| D | *SEEDSTICK* | *Nk*** | *OsMADS13* | *nk* | - | - | - |
| E | *SEPALLATA1, 2, 3*<br><br>*AGAMOUS-LIKE6* | *DEFH49, 72* | *LHS1, OsMADS5, 34* | *CsatSEP3*<br>*CsatAGL6* | 4<br>2 | famRCA-RACE<br>5′ 3′ RACE and famRCA-RACE | [39]<br>[21] |

*The number of crocus homologues for each type of MADs-box sequence obtained from crocus and method followed to obtain each crocus sequence is also indicated.

**nk: not known.

improvements of the method have been developed that consist of a collection of PCR-based cloning procedures that extend a known cDNA fragment toward the 3′ (3′ RACE) or the 5′ (5′ RACE) cDNA end. The original method is based on attachment of an anchor sequence to one end of the cDNA that can be used as a primer-binding template in a PCR with a second gene-specific primer from the known part of the gene.

As shown in Table 1, most of the original crocus sequences were obtained using the typical 5′ and 3′ RACE method. Since no ESTs are yet available from crocus flower for use as primers for the 5′ 3′ RACE, degenerate primers for conserved domains, specific for each type of crocus MADS-box type genes, were designed. For example, using 5′ 3′ RACE for obtaining *CsatAP3* sequences[27], a degenerate primer, corresponding to conserved amino acid sequence of the MADS-box genes, was used in 3′ RACE experiments with an dT Adaptor Primer. The synthesized cDNA was used as template in a touchdown PCR reaction with the degenerate primer and an Abridged Universal Amplification primer. Several products between 500 and 900 bp were cloned and sequenced, and based on the sequence information obtained by the 3′ RACE experiments, two gene-specific primers were designed from the 3-UTR and used to isolate the cDNA 5′ ends following the recommendations of the manufacturer[27]. For more details as to how the type of sequences using 5′ 3′ RACE were obtained, see the original publications for *CsatAP3*[27], *CsatAP1/FUL*[22] *CsatAG*[28], *CsatPI*[29], and *CsatAGL-6*[21]. For *CsatAP1/FUL*[22] and *CsatAG*[28], we used the abbreviation Cs as prefix to characterize the crocus genes, but since this caused some confusion because MADS-box genes from *Chloranthus spicatus* have been reported using the Cs prefix, we decided to use the Csat prefix for the crocus genes from now on and will update the GeneBank records, accordingly.

## The Development and Use of RCA-RACE Methods

The 5′ 3′ RACE method is time consuming and will take several years of work to identify the anticipated large number of MADS-box genes operating during crocus flowering and flower formation. Furthermore, 5′ 3′ RACE is technically difficult and usually requires substantial optimization and several repetitions before satisfactory results can be obtained[30]. Using a universal primer corresponding to the anchor sequence present in all cDNAs results in a high background of nonspecific products even after a nested PCR with a gene-specific primer internal to the first gene-specific primer is performed. Another drawback of the method is the difficulty of obtaining the full-length 5′ end of the transcript due to the presence of many truncated transcripts in the messenger RNA (mRNA) pool. Several strategies aimed at eliminating these problems have been developed, some of which require the generation of double-stranded cDNA, including the use of template-switching reverse transcription[31] or a postreverse transcription adaptor ligation step[32]. Methods that are performed directly on first-strand cDNA are complicated by the low efficiency of RNA ligase for the circularization reaction[33] or the need for bridging oligonucleotides for this step[34]. Furthermore, existing inverse-RACE methods typically require nested PCR to amplify the transcript of interest, and only a limited number of transcripts can be isolated from a single reverse transcription reaction, making it difficult to analyze rare transcripts from scarce tissue.

We have recently described an improved inverse-RACE method, named Rolling Circle Amplification – RACE (RCA-RACE)[35] (Fig. 1). The process takes advantage of the properties of CircLigase™ (Epicentre Biotechnologies, Madison, WI) to circularize single-stranded cDNA molecules via an intramolecular link for cDNA circularization, followed by rolling circle amplification[36] of the circular cDNA with φ29 DNA polymerase. In this way, a large amount of the PCR template is produced, allowing the simultaneous isolation of the 3′ and 5′ unknown ends of a virtually unlimited number of transcripts after a single reverse transcription reaction. To prove the concept, the method was used to isolate a previously characterized transcript: the crocus *AP1/FUL*-like MADS-box gene, which represents transcript coding for a rare transcript[27]. Subsequently, the method was also used to clone MADS-box genes from the peach (*Prunus percica*) fruit[37] and a number of genes from other crops.

We have also developed a modification of the RCA-RACE method named familyRCA-RACE (famRCA-RACE) that allows the simultaneous isolation of members of a family of homologous genes in one RCA-RACE reaction by using degenerate primers corresponding to a conserved amino acid domain in antisense orientation. To prove the concept of the proposed methodology, we used it to isolate members of the plant-specific family of NAC transcription factors sequences expressed in crocus flower[38]. The method was also used to obtain members of the MADS-box family of sequences from crocus. For obtaining the sequences of *CsatSEP3 (CsatSEP3A,B,C,D*), for example[39], and *CsatAP1/FUL* (*CsatAP1FULd,e*) using famRCA-RACE, first-strand cDNA synthesis on total RNA extracted from crocus flowers was purified, circularized, and amplified with φ29 DNA polymerase.

For obtaining MADS-box sequences out of this circular cDNA library, the φ29 amplified library was used as template in a PCR reaction with two degenerate primers, corresponding to conserved amino acid sequences of the MADS-box genes and designed in antisense orientation. Several individual clones were screened for the presence of an insert and sequenced giving MADS-box-containing inserts. Finally, based on the sequence information obtained from this famRCA-RACE experiment, gene-specific primers were designed and used for PCR to obtain the full-length coding sequences of the four *CsatSEP3*-like and *CsatAP1/FUL*-like genes mentioned in Table 1. Five additional clones with sequences identical to previously obtained sequences of *CsatAGL6a* and *CsatAGL6b* by 5′ 3′ RACE were also obtained, whereas experiments are underway to complete the sequence of one more *CsatAG*-like gene. The famRCA-RACE looks promising for eventually obtaining the full range of MADS-box sequences present in cultivated *Crocus sativus*. Following the same method and using the same degenerate primers, we can compare them with those of the putative progenitor diploid species.

## STRUCTURAL ANALYSIS OF THE CROCUS FLOWER MADS-BOX PROTEINS

All the organ-identity MADS-box genes isolated to date from crocus belong to type II MADS proteins, which are characterized by the distinct and highly conserved N-terminal MADS domain responsible for binding at DNA sequence elements known as CArG boxes[40]. According to Riechmann et al., this occurs

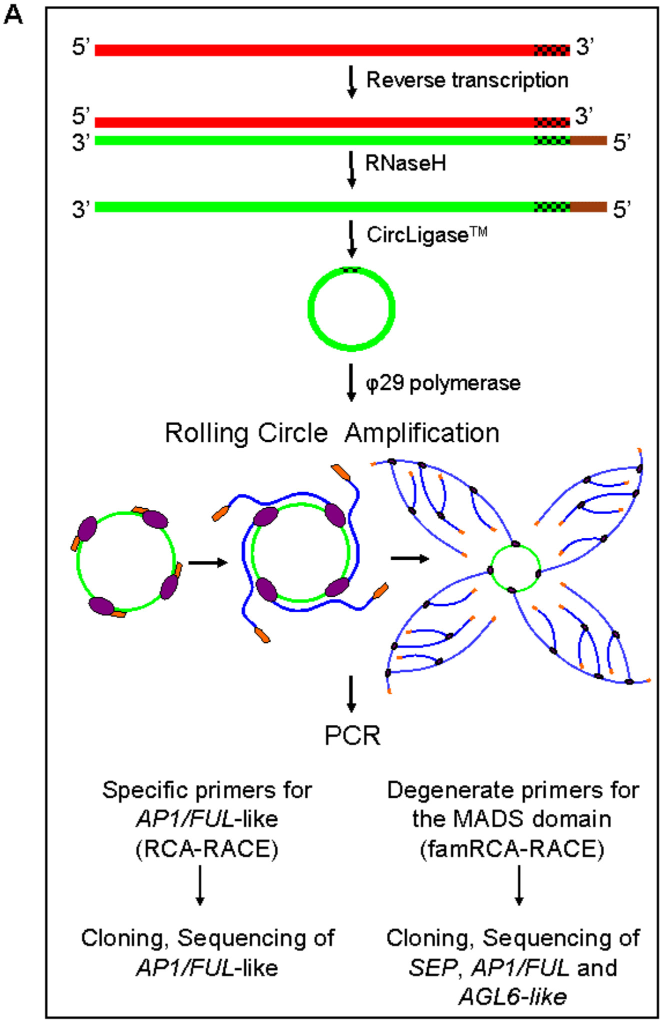

**FIGURE 1** Application of the RCA-RACE and the famRCA-RACE methods for obtaining crocus MADS-box cDNA sequences (see text for more details). (A) Illustration of the step taken to obtain the sequence of the crocus *CsatFUL/AP1a* gene, following the RCA-RACE method, and *CsatFUL/AP1d,e* and *CsatSEP3A,B,C,D* genes, following the famRCA-RACE method. Messenger RNA (mRNA; red) with a poly(A) tail (red checkerboard pattern) is reverse transcribed into cDNA (bright green) using an oligo(dT) (bright green checkerboard pattern) primer harboring a 5′ phosphorylated adaptor (brown). After RNaseH treatment, the resulting cDNA is circularized using CircLigase. The circular cDNA is then amplified by RCA using φ29 DNA polymerase (violet oval) and random hexamer primers (orange) to multicopy concatemers (blue). For each transcript of interest, an aliquot of the RCA reaction serves as a template in an inverse PCR with specific or degenerate primers to obtain the transcript's 5′ and 3′ ends simultaneously. (B) Amplification products of *CsatFUL/AP1a* transcript using CsatAP1-F/Inv1 primers on serial dilutions of RCA reactions performed with the InVUP primer (lanes 1–4) or random hexamers (lanes 5–8). Lanes 1 and 5, $10^{-1}$ template dilution; lanes 2 and 6, $10^{-2}$ template dilution. In this experiment, control PCRs were performed with template produced in RCA, in which φ29 was omitted from the reaction to test if circularized, but not amplified, single-stranded cDNA could provide a suitable template for PCR. Lanes 3 and 4 represent the controls for lanes 1 and 2, respectively; similarly, lanes 7 and 8 are the controls for lanes 5 and 6, respectively. M indicates the λ*Hin*dIII/ΦX174*Hae*III molecular weight marker, and relevant sizes in base pairs are shown at the right. (C) Hybridization of the PCR products after transfer to a membrane with the *CsatFUL/AP1a* probe. The visible gel bands (lanes 5 and 6), as well as a single band of the expected size (approximately 850 bp) not visible on the gel (lane 1) gave hybridization signals. (D) Electrophoresis of famRCA-RACE reactions performed for the MADS-box subfamily of *SEP*-like genes of crocus using MADS-2F/MADSPromR degenerate primers on 1 µl of the initial φ29 reaction with an InVUP primer (lane 1) and $10^{-1}$ dilution (lane 2). M indicates the 100-bp molecular weight marker, and relevant sizes in base pairs are shown at the right.

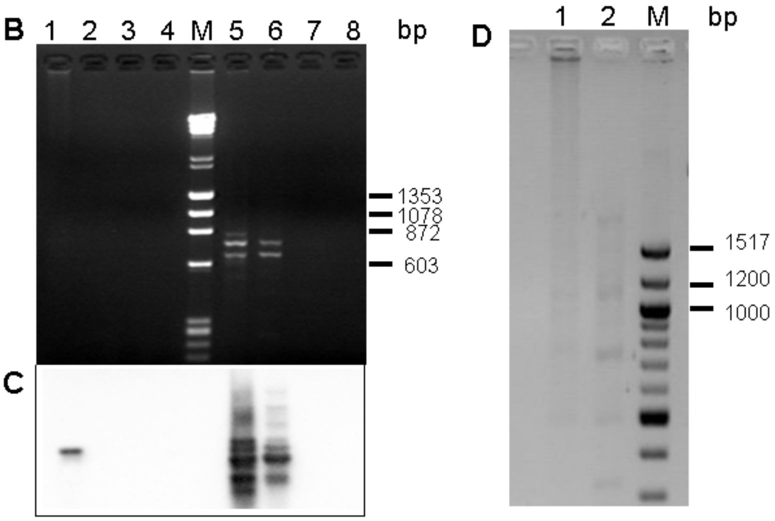

**FIGURE 1B–D**

only when the MADS transcription factor proteins are dimerized, which is primarily mediated by the adjacent I and K dimerization domains, also preserved in all crocus MADS proteins isolated. On the contrary, the C domain on the carboxyl protein terminal is relatively more variable in both the length and the sequences involved, but lineage-specific, short, highly conserved sequence motifs are also present in the C domain indicative of its significance functional specificity[41]. Alignments constructed using *Clustal W*[42] of the C-terminal motifs that are characteristic for deep branching of the MADS proteins in each lineage are collectively shown in Fig. 2 for all the crocus MADS proteins isolated, together with homologues plant MADS proteins. For each lineage of the MADS proteins, characteristic C-terminus motifs are boxed according to Vandenbussche et al.[43] and Kramer et al.[44], while the crocus sequences are in bold.

More specifically, as shown in Fig. 2, the C-terminal domain of AP1/FUL predicted proteins is highly variable, as is characteristic of plant MADS-domain-containing proteins. Nonetheless, there is a strongly conserved hydrophobic six-amino-acid motif (consensus LPPWML) at the end of all FUL-like and euFUL proteins. This FUL-like motif can be seen in SEP and AGL6 sequences, although the exact residue composition is not strictly conserved. The high degree of conservation of this motif is a strong indication that it is functionally important and suggests that its loss and replacement with a different motif in euAP1 proteins may result in altered functional capabilities of the euAP1 proteins[45]. Published proteins belonging to the AP1/FUL lineage, which showed a high degree of homology to the crocus CsatFUL/AP1, were selected for the multiple alignment process as previously described[27]. In this comparison, the three crocus homologues A, B, C presented 87, 82, 84% identity and 92, 92, 91% similarity to the consensus FUL/AP1-like proteins MADS-box domain, and 64, 59, 51% identity and 79, 77, 76% similarity to the consensus K-box of the AP1/FUL -like proteins, respectively. High identity and similarity percentages were calculated between all the three homologues and the maize ZmM28 and the rice OsMADS15 proteins. The amino acid sequence of *AP1* in dicots has a characteristic EuAP1 C-terminal motif and many dicot homologues terminate in CFAA, a typical CaaX box recognition motif for farnesyltransferase (FTase). *AP1* is a target of FTase and farnesylation alters the function and perhaps specificity of the transcription factor[46]. The five crocus *AP1/FUL*-like genes lack a CaaX box at the C-terminus, since monocot genes in this lineage terminate with a paleoAP1 motif characteristic for dicot *FRUITFULL* (*FUL*) genes (previously called *AGL8* in *Arabidopsis*). For this reason, we also decided to rename the previously published *CsatAP1/FUL*[27] to *CsatAP1/FUL*. *Arabidopsis FUL* is weakly expressed in rosette leaves during vegetative development and is subsequently strongly up-regulated in the shoot apex on the transition to flowering. Experimental evidence suggests that *FUL* regulates the transcription of genes required for cellular differentiation during fruit and leaf development[16].

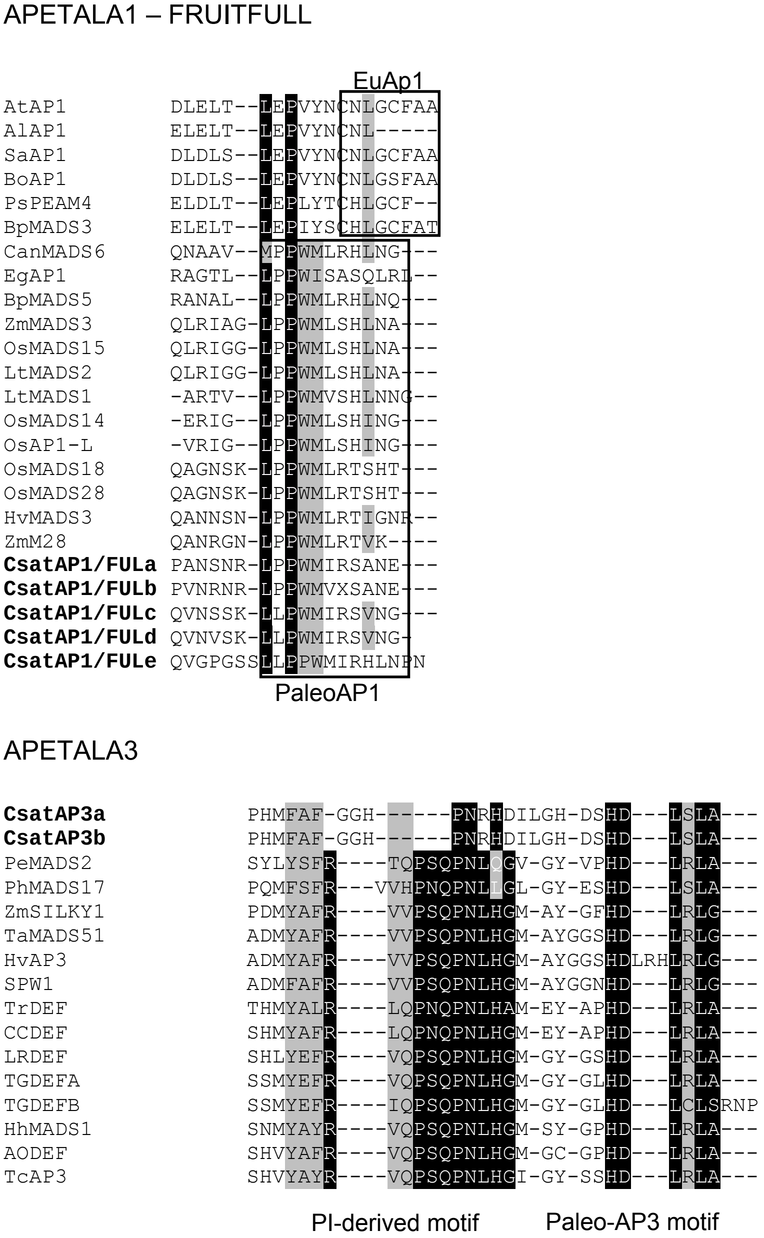

**(continues)**

**FIGURE 2** Alignment of the deduced amino acid (aa) sequences of the C-terminus of crocus MADS-box genes and other homologous plant MADS proteins constructed with *Clustal W*[42]. Identical amino acids are highlighted in black and similar amino acids in gray with a threshold for shading of 70% sequence similarity. For each lineage of the MADS-box proteins, characteristic motifs are boxed. Motifs are described in Vandenbussche et al.[43] and Kramer et al.[44]. Crocus sequences are indicated in bold (see text for details).

*AP3*-like genes in higher plants have conserved C-terminal motifs, which in most higher eudicots is called the eu*AP3* motif, and in lower eudicots and magnoliids is called the paleo*AP3* motif[41]. The ancestral paleoAP3 motif was retained in the so-called TM lineage of paralogous core eudicot lineages[47]. Detailed comparison between the paleo*AP3* and the eu*AP3* motif revealed that the latter could have been derived by an 8-bp insertion at the C-terminus of the former, causing a frame shift mutation beyond the insertion site in eu*AP3* genes[43]. The paleoAP3 motif is also conserved in many B-class AP3-like proteins in monocots, so far examined including lily, asparagus, and tulip[48,49,50]. This led to the suggestion that all monocot AP3-like proteins have a paleoAP3 motif[50], which is in agreement with recent hypotheses

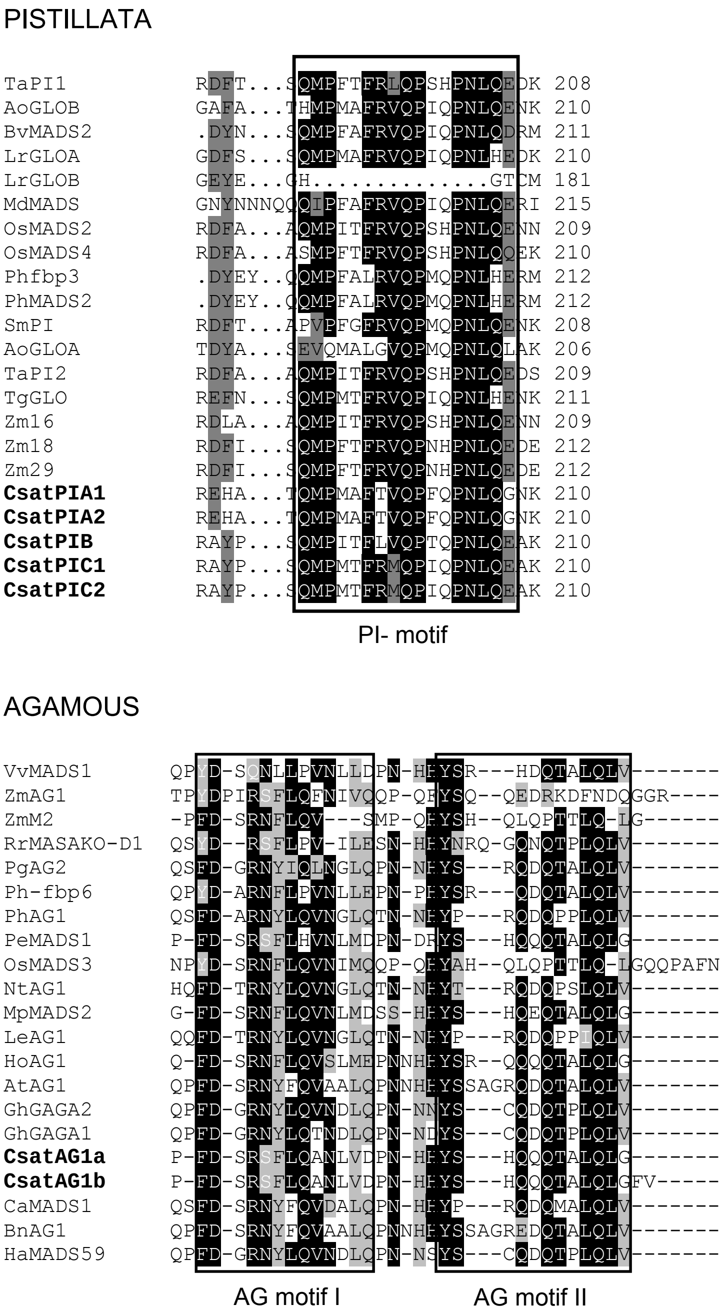

(continues)

**FIGURE 2 continued**

on the phylogenetic position of monocots in basal angiosperms[51]. This hypothesis is supported by the presence of the paleoAP3 motif in the crocus AP3-like sequences (five out of eight identical amino acids with the consensus paleoAP3 sequence [52]). Another conserved motif in AP3-like proteins is the PI-derived motif, which is defined as a region bearing similarity with the conserved PI motif in the PI lineage of B-class proteins[41]. This region is less conserved in all AP3-like lineages including the paleoAP3 lineage of monocots, and the crocus sequence has only five out of 12 identical amino acids of the consensus PI-derived motif described previously[52]. Alignment of the predicted amino acid sequences CsatAP3a and CsatAP3b with the members of B-class MADS-box proteins used in phylogenetic analysis[27] revealed that

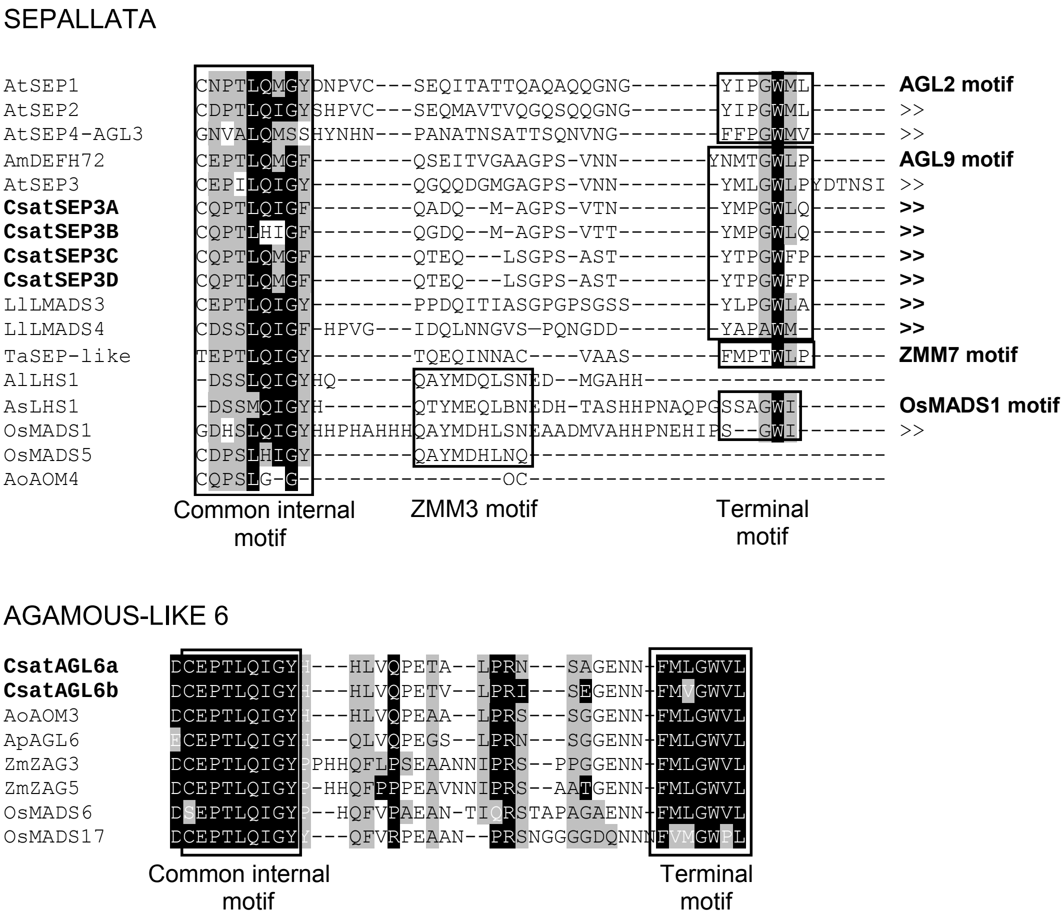

**FIGURE 2 continued**

the crocus sequences share high similarity with B-class genes in the conserved MIK region whereas they are more divergent in the variable C region. Within the C region, the paleoAP3 motif in the crocus AP3-like sequences (five out of eight identical amino acids with the consensus paleoAP3 sequence described in Tzeng and Yang[52]) could be identified. The PI-derived motif, which is defined as a region bearing similarity with the conserved PI motif in the PI lineage of B-class proteins[47] was less conserved (five out of 12 identical amino acids of the consensus PI-derived motif described in Tzeng and Yang[52]).

Based on the amino acid sequence similarity of the entire coding region, the five CsatPI homologous-deduced protein sequences can be assigned to the PI/GLO-like family of proteins[53]. Published genes that belong to the PI/GLO family of proteins and showed high degree of homology to the five CsatPI were selected for multiple alignment[29]. All five CsatPI proteins at the C-terminus include a PI-motif, which is conserved sequence in the *PI/GLO* family genes[47].

C-type MADS-box genes homologous to *AGAMOUS* (AG) from crocus revealed two transcripts designated *CsatAG1a* and *CsatAG1b*, which are different since *CsatAG1a* is missing 10 bp from 890 to 899 in comparison with *CsatAG1b*. This deletion alters the coding ORF of *CsatAG1a* in such a way that the deduced protein sequence of *CsatAG1a* is two aa shorter at the C-terminus from the deduced protein sequence of *CsatAG1b*. Alignment of C-terminal regions of predicted amino acid sequences for selected representatives of the C and D lineages and gymnosperm (Gymno) *AG*-like genes distinguished two highly conserved regions, AG motif I and AG motif II[44]. Both crocus transcripts contain an identical AG motif I and their AG motif II have the above described two aa difference at the 3′ end.

SEP genes form a well-supported clade within the MADS-box gene phylogeny that can be further subdivided into three clades: a mixed eudicot and monocot clade containing the *Arabidopsis SEP3* gene matching all isolated crocus SEP sequences; a eudicot clade containing the *Arabidopsis SEP1, SEP2*, and *AGL* genes; and a clade comprised solely of monocot sequences[54]. All lineages have a conserved C-terminus internal motif in common, but diverse motifs at the 3′ end. The alignment of CsatSEP3-deduced amino acid C-terminal sequences with other SEP-like proteins revealed a C-terminal motif (YMPGWLQ) typical for members of the SEP3 lineage[43] present in CsatSEP3A and CsatSEP3B, while a similar, but modified (YTPGWFP), motif was present in CsatSEP3C and CsatSEP3D sequences. Similar motifs are present in *Arabidopsis* SEP3, *Antirrhinum* EFH72, and *Lilium longiflorum* LMADS3 and LMADS4.

The alignment of the two CsatAGL6-deduced amino acid sequences with other monocot AGL6-like proteins revealed a number of conserved L amino acids that play a role in protein-protein interactions as observed in the K domain[21]. There are also two conserved motifs at the C-terminus domain that are found in AGL6-like proteins: the FMLGWVL motif, typical for the AGL-6 subfamily[43] and a CEPTLQIGYH motif found in the AGL6 subfamily and in the ZMM7 lineage of the SEP subfamily of MADS-box proteins[43].

## PHYLOGENIC COMPARISONS OF CROCUS MADS-BOX SEQUENCES

MADS-box genes in plants comprise a large family, counting 107 genes in *Arabidopsis*[55], 71 genes in rice[17], and probably numerous members in other plants. They are also present in nonflowering plants such as gymnosperms, ferns, and mosses, indicating that their role is not restricted to flower development. Phylogenetic relationships of plant MADS-box genes suggest that the different clades were probably established by gene duplication, diversification, and fixation. Genes within each clade acquired specific functions during the evolution of flowering plants[56].

The crocus sequences thus far isolated by our group sum up to 20 cDNAs, representing five major clades of the MADS phylogeny and the different A, B, C, and E functions of these genes. In Fig. 3, the phylogenetic tree showing the relationship of the crocus MADS-box sequences with related monocot members is presented. For constructing the tree, the Neighbor-Joining method was employed. The topology of the tree is in agreement with published topologies of angiosperm MADS-box proteins[14]. In this tree, the AP1/FUL, SEP, and AGL6 clades converge on a common node. This branch is always found in MADS phylogeny reconstructions[14] and is connected with the AG clade. The AP3 and PI clades also share a common node. Placement of the homologous genes in each clade is supported with high values in bootstrap resampling. The topology of the crocus MADS-box tree reflects the relationships between members of the major clades of MIKC-type genes present in angiosperms.

While core eudicots like *Arabidopsis*, *Antirrhinum*, *Petunia,* and others provided important information concerning the phylogenetic relationships of MADS proteins, comparisons with monocots like grasses and nongrass monocots like crocus represent a much longer evolutionary divergence of at least 130 mya[57]. The analysis with crocus indicated that crocus contains representatives of all ancestral gene lineages predating the core eudicot dublications[58], such as the *CsatAP1/FUL*, *CsatpaleoAP3*, *CsatAG,* and *CsatSEP3* genes. In comparison to grass monocots like rice and maize, our data indicate that crocus only contains the ancestral *SEP3*-like gene, as all four sequences obtained are highly homologous to *SEP3*, while grasses like rice and maize apparently underwent separate SEP lineage proliferation since they contain SEP2, 3, and 4 homologues[54].

## FUTURE PERSPECTIVES

Further experiments are underway for isolation of the complete set of crocus MADS-box genes, and elucidation of their role in crocus flower development and tepal formation, but also possible roles in other aspects of crocus physiology like transition to flowering, corm formation, etc. For this goal, the famRCA-

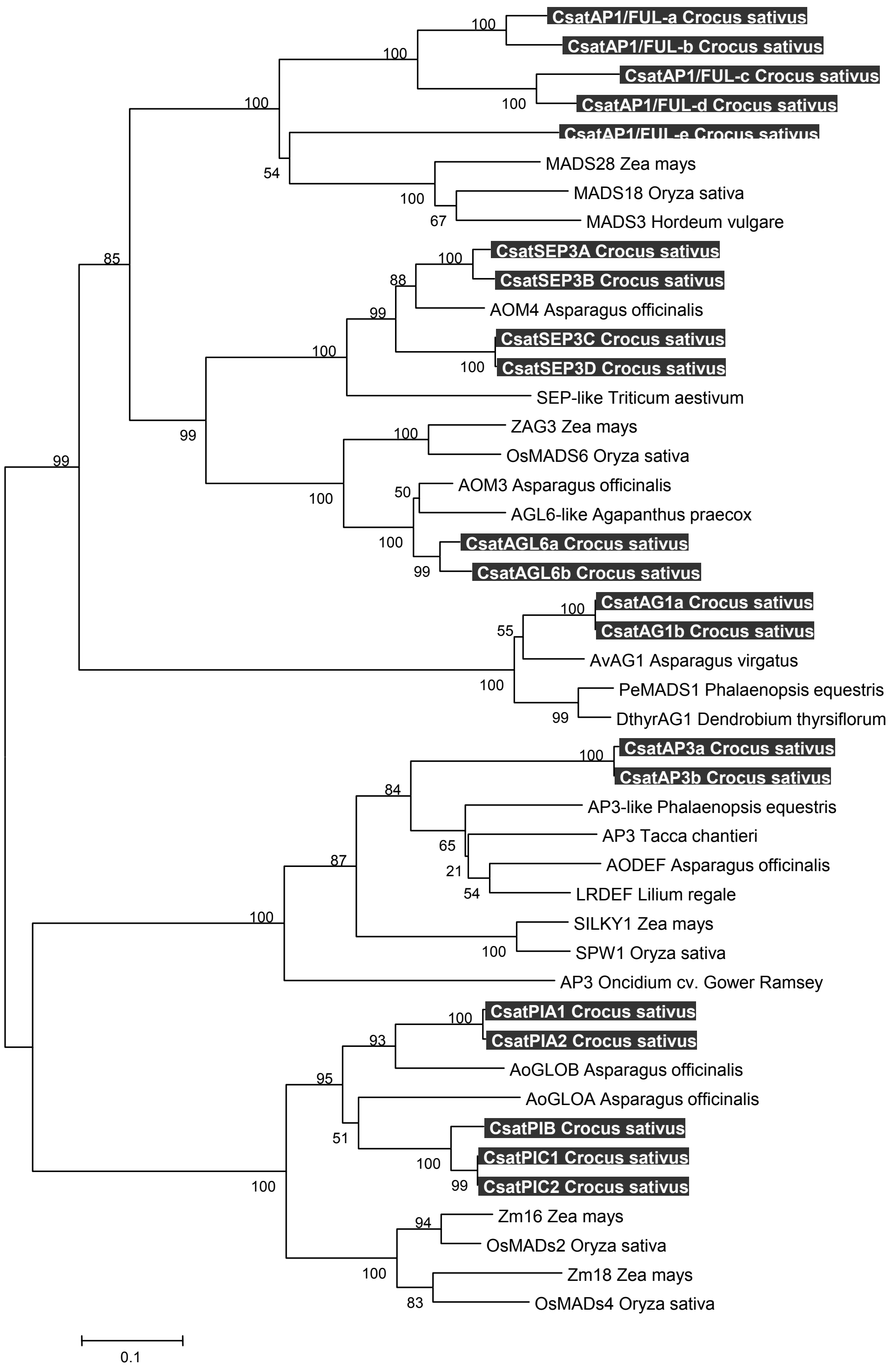

**FIGURE 3** Phylogenetic relationships of MADS-box proteins of crocus and related monocots. Amino acid sequences were aligned with *Clustal W*[42]. The tree was generated by the Neighbor-Joining method using the p-distance correction. Numbers next to the nodes are bootstrap values from 1000 replications. The scale indicates amino acid substitutions.

RACE method described in this review, which allows the simultaneous isolation of members of a family of homologous genes, may prove to be a very useful and highly efficient tool. Furthermore, the availability of a complete set of MADS-box sequences from the cultivated *C. sativus* triploid species, forms the base for similar analysis of this and other important families of proteins studied[38] in the putative progenitor diploid species of cultivated crocus. Comparative structural and phylogenetic analysis of these proteins will be helpful to solve the origin of the cultivated triploid *C. sativus*. Finally, it will be possible to characterize the numerous field isolated flower mutants of *C. sativus* for further elucidating the role of the different groups of the A-, B-, C-, and E-type MADS proteins in crocus flower organ formation.

## ACKNOWLEDGMENTS

The authors are grateful to Mr. Yiannis Patsios for his invaluable help in collecting plant material in the field, and to all of our lab members for helpful comments and discussion. Work at the Institute of Agrobiotechnology is supported by the General Secretariat for the Research and Technology (Greek Ministry of Development).
